# Binding of Phosphate Species to Ca^2+^ and Mg^2+^ in Aqueous Solution

**DOI:** 10.1021/acs.jctc.4c00218

**Published:** 2024-05-08

**Authors:** Basak Koca Fındık, Majid Jafari, Lin Frank Song, Zhen Li, Viktorya Aviyente, Kenneth M. Merz

**Affiliations:** † Department of Chemistry, Bogazici University, Bebek, Istanbul 34342, Turkey; # Department of Biochemistry and Molecular Biology, Michigan State University, East Lansing, Michigan 48824, United States; § Biochemical and Biophysical Systems Group, Lawrence Livermore National Laboratory, Livermore, California 94550, United States; ‡ Department of Chemistry, Michigan State University, East Lansing, Michigan 48824, United States

## Abstract

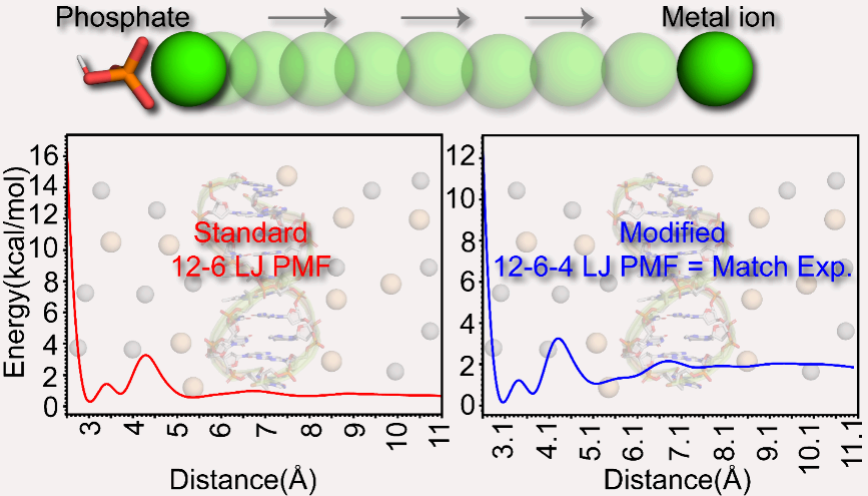

Phosphate derivatives and their interaction with metal cations are involved in many important biological phenomena, so an accurate characterization of the phosphate-metal interaction is necessary to properly understand the role of phosphate-metal contacts in mediating biological function. Herein, we improved the standard 12-6 Lennard-Jones (LJ) potential via the usage of the 12-6-4 LJ model, which incorporates ion-induced dipole interactions. Via parameter scanning, we fine-tuned the 12-6-4 LJ polarizability values to obtain accurate absolute binding free energies for the phosphate anions H_2_PO_4_^–^, HPO_4_^2–^, PO_4_^3–^ coordinating with Ca^2+^ and Mg^2+^. First, we modified the phosphate 12-6-4 LJ parameters to reproduce the solvation free energies of the series of phosphate anions using the thermodynamic integration (TI) method. Then, using the potential mean force (PMF) method, the polarizability of the metal-phosphate interaction was obtained. We show that the free energy profiles of phosphate ions coordinated to Ca^2+^ and Mg^2+^ generally show similar trends at longer metal–phosphate distances, while the absolute binding energy values increased with deprotonation. The resulting parameters demonstrate the flexibility of the 12-6-4 LJ-type nonbonded model and its usefulness in accurately describing cation–anion interactions.

## Introduction

Organo-phosphate species are among the most abundant chemical groups in nature, and more than three decades ago, Westheimer provided a detailed analysis of the essential role of phosphate in living systems.^1^ More critically, in the cases of DNA or RNA, the ionic structure of phosphates allows the binding of two nucleosides by forming a phosphodiester bond, while the negative charges are located outside of the backbone. They are stabilized by metal ion coordination or water molecules, and the binding of divalent metal ions leads to conformational changes while determining their tertiary structures.^2−7^ Also, nucleoside phosphate anions such as ATP, ADP, GTP, and UDP play key roles in DNA synthesis, energy transduction, and cellular signaling, and they are accompanied by divalent metal ions, most commonly Mg^2+^, coordinated to the phosphate group. Apart from cell metabolism, phosphates are also found in the outer layer of bone, consisting mainly of hydroxyapatite, a Ca^2+^-coordinated phosphate mineral.^8,9^ Phosphates are also found in the polar part of phospholipids; they form the building blocks of cell membranes, and coordination of Mg^2+^ and Ca^2+^ to phosphates causes partial dehydration, conformational changes, and phosphodiester group immobilization.^10^

Phosphate derivatives, namely H_2_PO_4_^–^, HPO_4_^2–^, and PO_4_^3–^, are present as ionized species within the physiological environment due to their p*K*_*a*_ values from H_3_PO_4_ (p*K*_*a*_ = 2.2, 7.2 and 12.3, respectively). Among them, HPO_4_^2–^ and H_2_PO_4_^–^ are the most abundant forms in solution at physiological pH. The anionic nature of these ions adds hydrophilicity while also providing coordination sites to metal ions in biological processes.

Elucidation of the atomic interactions of metal ions with biomacromolecules provides an understanding of their structure–function relationships in biological systems.^11^ While a quantum mechanical explanation of these phenomena would be highly desirable, the size of biological systems sets a limit because of the computational expense. Force field methods are used to overcome this issue; however, metal ions have high polarizabilities, which renders the inclusion of metal ions into force field methods challenging.^11−13^ In addition, the nature of a force field directly affects the efficiency of predicting the thermodynamic and structural properties of metal bonding systems.^12^ To reach this goal, significant efforts have been invested in developing force field models for classical modeling over the years.^11,13−21^ A commonly used approach to model metal–ligand interactions is the conventional nonbonded Lennard-Jones (LJ) (widely known as 12–6 LJ) in which the divalent metals are represented as point charges. In the relevant literature, many articles focus on the development of parameter sets that are tuned against experimental properties such as hydration free energies (HFE), solvent–ion radial distribution functions (RDF) and ion-oxygen distances (IOD) in the first water coordination shell.^11^ However, it is difficult to reproduce experimental properties simultaneously since the conventional nonbonded Lennard-Jones model suffers from the lack of polarizability: Therefore, its applicability is limited. To overcome this drawback, Li and Merz have proposed a 12–6–4 LJ nonbonded model, which includes an additional 1/r^4^ term to the standard 12–6 LJ potential, the latter taking care of induced dipole interactions.^22^ The 12–6–4 model is computationally efficient, easy to apply, and after careful parametrization, the resultant parameter sets can be transferred to macromolecular systems.^13,22−24^

The aim of this work is to parametrize the polarizability (α) values of the 12–6–4 LJ potential to describe the interactions of phosphate derivatives with divalent metal cations, while elucidating the interactions of these species with divalent metals. Due to their biological importance, Ca^2+^ and Mg^2+^ ions were selected for the study of their interactions with H_2_PO_4_^–^, HPO_4_^2–^ and PO_4_^3–^ in aqueous solution. Using available experimental data,^25−27^ we obtained accurate models for the HFE of the anions in aqueous solution (using TI), which were then used to develop parameters for Ca^2+^-phosphate and Mg^2+^-phosphate interactions via the umbrella sampling method.

## Computational Details

In the present work, the 12–6–4 LJ potential has been used along with the AMBER force field (eq 1)
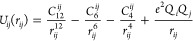
1in which *C*_12_^*ij*^/*r*_*ij*_^12^ is a repulsive term that prevents the attraction from becoming too strong at close distances, *C*_6_^*ij*^/*r*_*ij*_^6^ is an attractive term derived from dispersion forces, *C*_4_^*ij*^/*r*_*ij*_^4^ is an additional attractive term which considers the polarization of charged species (α_0_) (eq 2). The last term of eq 1, *e*^2^*Q*_*i*_*Q*_*j*_/*r*_*ij*_, represents the Coulombic interactions where *e* is the charge of the proton and *Q*_*i*_ and *Q*_*j*_ are partial charges of atoms *i* and *j*.
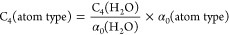
2

The C_4_ terms between water and ions were parametrized in earlier studies.^13,19,20,22,28,29^ For this reason, potential mean force (PMF) calculations were performed to parametrize the C_4_ term values of the interactions of phosphate derivatives with Ca^2+^ and Mg^2+^. In PMF calculations, the C_4_ term belonging to water-Ca^2+^ and water-Mg^2+^ interactions were taken from previous work.^22^ However, phosphate derivatives are also in ionized forms in the aqueous solution, and their interactions with water are also considered through a polarizability value parametrized against experimental hydration free energy (HFE) values via Thermodynamic Integration (TI) calculations.

### System Preparation

A geometry optimization of phosphate derivatives was performed at the MP2^30−33^/6-311+G(d,p) level of theory in aqueous solution using the implicit solvation model SMD^34^ implemented in Gaussian 16.^35^ The charge values calculated with the CM5 model^36^ were introduced in the mol2 file generated using the Antechamber program.^37^ The mol2 files used for PMF calculations are provided in the Supporting Information (SI). The LEaP module was used to generate the topologies using the generalized AMBER force field 2^38^ (GAFF2) for phosphate derivatives. All molecular dynamics simulations were conducted via the CUDA version of PMEMD from the AMBER22^39^ package. The VMD program^40^ was used to visualize the trajectories.

### Hydration Free Energy Calculations

**Figure 1:**
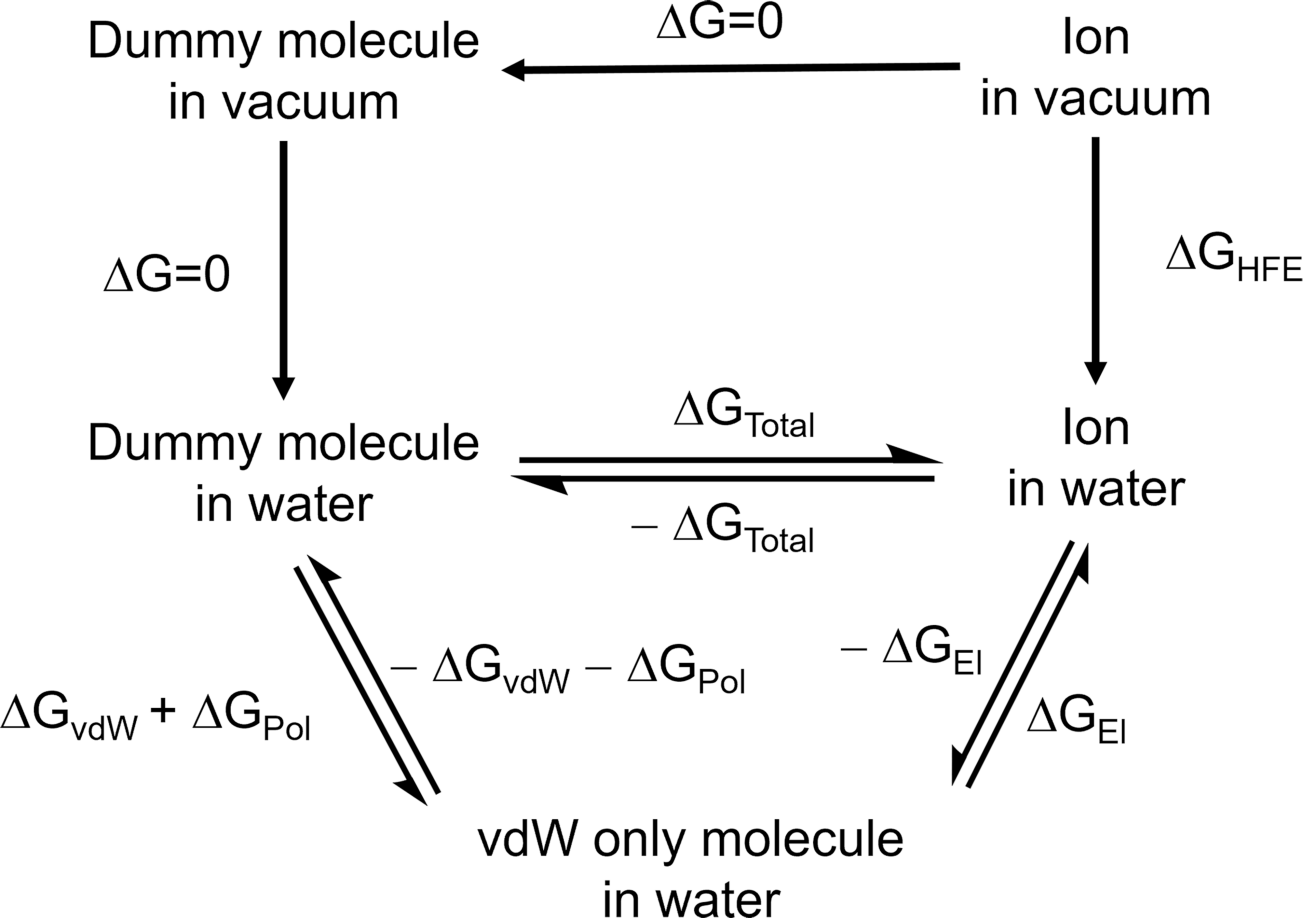
Thermodynamic cycle for one-step and two-step protocols for calculating the HFE values.

The calculation of hydration free energy (HFE) involves using the concept of a thermodynamic cycle in Figure 1, where the sum of free energy changes around a closed cycle is zero due to the free energy being a state function. This protocol has been used extensively in our previous studies.^12,13,19,20,22,28,41^ The HFE can be calculated by following the process of moving a molecule from the gas phase to the aqueous phase following two different cycles, using the one-step protocol and two-step protocol. In the one-step protocol, the van der Waals, polarizability (represented by C_4_ term in aqueous phase calculations), and electronic parameters are scaled together along the alchemical path (Δ*G*_total_ in Figure 1), whereas in the two-step protocol, all force field parameters (Δ*G*_vdW_, Δ*G*_Pol_ and Δ*G*_Ele_) are scaled separately. To avoid the “end-point catastrophe” during the Δ*G*_vdW_ calculations, the soft-core potential protocol employs a modified LJ potential changing with the λ value (eq 3), where α is 0.5 and σ is the distance at which LJ potential between atoms (i and j) is equal to 0.
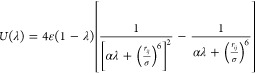
3

The free energy difference between two states (gas (A) to water (B)) was calculated by dU(λ)/dλ integration where U(λ) is the potential function while λ is the coupling parameter (eq 4). Gaussian quadrature (eq 5) is used to evaluate the integral efficiently.
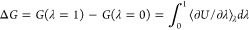
4
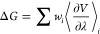
5

Each phosphate species was solvated in a cubic box (39 × 39 × 39 Å^3^) together with their corresponding dummy atom versions, surrounded by TIP3P^42^ water molecules. Twelve independent λ runs were performed (0.00922, 0.04794, 0.11505, 0.20634, 0.31608, 0.43738, 0.56262, 0.68392, 0.79366, 0.88495, 0.95206, 0.99078) and their average values were reported. For each λ, of each alchemical pathway, 20000 steps of steepest descent minimization were performed, followed by a heating step in the NVT ensemble, in which the system was gradually heated up to 300 K at the ps time scale. To equilibrate the density, the equilibration step was performed in the NPT ensemble at 300 K using the Monte Carlo Barostat^43^ for 1 ns, followed by a 4 ns production run in which dU(λ)/dλ data was collected. For all TI simulations, SHAKE was used, and the time step was 1 fs. Note that for gas phase calculations, the nonbonded interaction cutoff value was set to 999.

To account for species without experimental HFE values, i.e. HPO4^2–^, the HFE values of each phosphate derivative were calculated at the M06^44^/6-31++G(d,p) level of theory using the SM8 solvation model^45^ with the dual basis approximation. For this purpose, the Q-Chem 5.4 software package^46^ was used and with the help of the extrapolation of QM-calculated and FF-calculated HFE values, the experimental HFE value has been estimated. See Results and Discussion for more details.

### Binding Free Energy Profiles

Each phosphate derivative coordinated with a divalent metal ion was solvated in a 59 × 59 × 59 Å^3^ TIP3P cubic box, and the distance between the metal ion and the phosphorus atom of the phosphate species was constrained to 11 Å as the starting structure for subsequent umbrella sampling (US) simulations. For the metal ions, the 12–6–4 LJ non bonded model has been used. 10000 steps of steepest descent minimization were performed, followed by 10000 steps of conjugate gradient minimization. Afterward, the system was gradually heated from 0 to 300 K over 200 ps using the canonical ensemble, followed by 2 ns NPT equilibration and 8 ns NPT production at 300 K. The weighted histogram method (WHAM) was used to generate the energy profile with respect to the reaction coordinate. The Berendsen barostat was used for pressure control, and the Langevin thermostat was used to maintain a constant temperature with a collision frequency of 1 ps^–1^. The restraint constant for each window was tuned to ensure that the sampled distances are distributed around the targeted value and that neighboring windows overlap. The US-calculated binding free energies were ±0.30 kcal/mol within the experimental binding value. The Particle-Mesh-Ewald (PME) method was used to treat the long-range electrostatic interactions and a 12 Å cutoff was used for nonbonded interactions.

## Results and Discussion

### Analysis of the Results from the Optimized 12-6-4 LJ Parameter Sets

Before examining metal ion binding, the C_4_ terms of the ionized phosphate derivatives were tuned. In 2012, Case et al. obtained the parameter set for phosphate derivatives via the Pearson cycle by manipulating the van der Waals radii of phosphate oxygens to shield the electrostatic interactions with water.^47^ As an alternative approach, we have parametrized the 12–6–4 nonbonded LJ potential combined with US and TI calculations to optimize the interactions of the phosphate ions in water and their interactions with Ca^2+^ and Mg^2+^.

The calculated HFE values, experimental HFE values and corresponding C_4_ values between deprotonated phosphate oxygen atoms and water are tabulated in Table 1. The experimental HFE values of Marcus^25^ were targeted to be consistent with previous studies.^22,23,48^ Marcus reported the HFE, hydration enthalpy, and entropy values for a set of ions, including H_2_PO_4_^–^ and PO_4_^3–^ species. However, the experimental values of the hydration free energies of other phosphate ions, in particular, HPO_4_^2–^, are not available at this point. For this reason, the hydration free energies of HPO_4_^2–^, H_2_PO_4_^–^, and PO_4_^3–^ species were calculated at the M06/6-31++G(d,p) level of theory employing the SM8 solvation model. Later, QM-calculated HFEs of H_2_PO_4_^–^ and PO_4_^3–^ were extrapolated with the corresponding experimental values and the experimental HFE of HPO_4_^2–^ was approximated via a linear regression approach as reported in Table S1 and the extrapolation equation used to obtain the missing value is *y = 1.0763 x–22.984*. The TI-calculated Δ*G*_HFE_ values for both protocols are consistent with the experimental results, and the obtained values are within the range of ±0.05 kcal/mol of experiment.

**Table 1:** Experimental, QM-Calculated and FF-Calculated Δ*G*_HFE_ Values (kcal/mol) for each Phosphate Derivative^a^

**Phosphate Derivatives**	**C**_**4**_**between o and OW**	**Experimental Δ***G*_**HFE**_	**FF- calculated Δ***G*_**HFE**_
H_2_PO_4_^–^	73	–111.06	–111.11
HPO_4_^2–^	22	–315.69^b^	–315.64
PO_4_^3–^	–18	–660.85	–660.92

a The deprotonated oxygen atom of phosphate and water oxygen are denoted as o and OW respectively.

b Extrapolated value.

The binding interactions of phosphate derivatives with Ca^2+^ and Mg^2+^ were parametrized against the experimental association constants (*K*_*a*_) taken from Chungtai et al.^27^ and Verbeeck et al.,^26^ respectively. The experimental binding free energies (Δ*G*_bind_) were calculated in kcal/mol by using eq 5 in which T is the temperature (298 K) and R is the universal gas constant.
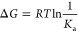
6

**Figure 2:**
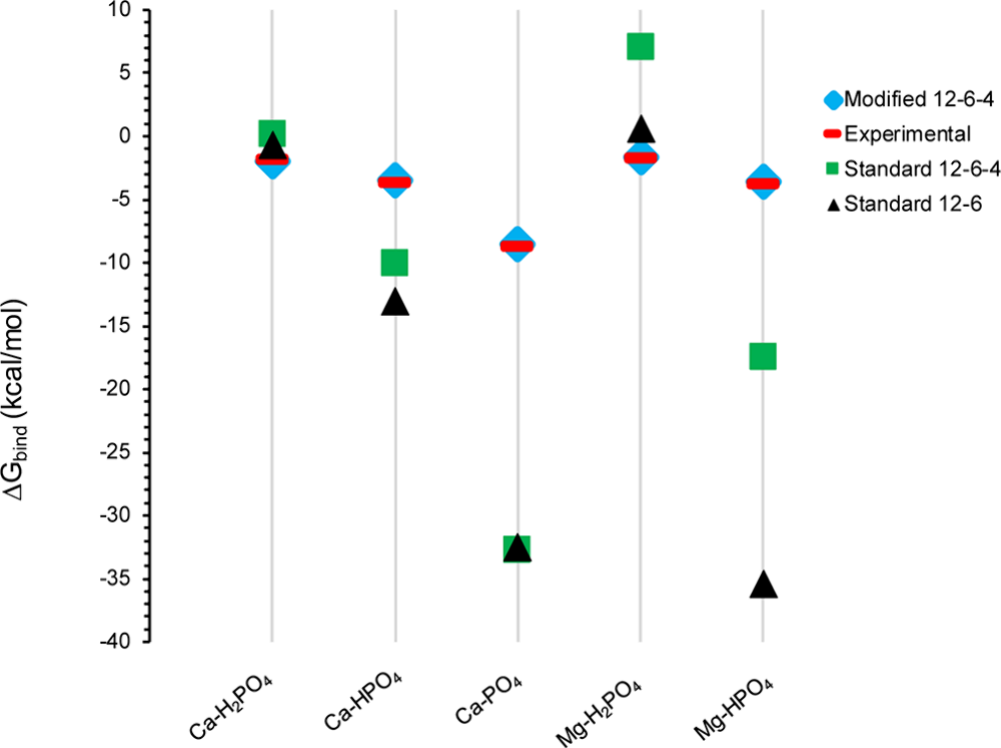
Comparison of binding energies calculated using both the standard 12-6-4 values and the standard 12-6 values, alongside the 12-6-4 modified values parametrized in this study. For detailed energy values and further information, see Table S2.

The experimental binding free energies are given in Table 2, together with the calculated free energies obtained with modified C_4_ values of the 12–6–4 LJ potential. According to the experimental findings, the interaction of phosphate derivatives with both Ca^2+^ and Mg^2+^ becomes more favorable when the negative charge on the phosphate derivative increases. The results obtained in the binding energy study with the default values for 12–6–4 and 12–6 are quite different from the experimental values (Figure 2). It was observed that as the negative charge on the phosphate species increases, the deviation from the experimental value increases. Additionally, it was observed that the unbounded complex structure was more favorable relative to the bound complex for the H_2_PO_4_^–^ coordinated systems.

**Table 2:** Experimental and Calculated Δ*G*_bind_ Values (kcal/mol), Together with the Refined C_4_ Term of 12-6-4 LJ Potential Which Were Parametrized against Experimental Values^a^

			**Calculated Δ***G*_**bind**_				
**Divalent Metal**	**Phosphate Derivative**	**Experimental Δ***G*_**bind**_	**Rep #1**	**Rep #2**	**Rep #3**	**Average ± std**	**C**_**4**_**between M**^**2+**^**-o**
Ca^2+^	H_2_PO_4_^–^	–1.92	–1.89	–1.97	–2.02	–1.96 ± 0.07	104.00
	HPO_4_^2–^	–3.70	–3.54	–3.47	–3.61	–3.54 ± 0.07	–69.50
	PO_4_^3–^	–8.81	–8.69	–8.34	–8.56	–8.53 ± 0.18	–257.00
Mg^2+^	H_2_PO_4_^–^	–1.73	–1.75	–1.65	–1.73	–1.71 ± 0.05	174.90
	HPO_4_^2–^	–3.86	–3.63	–3.52	–3.49	–3.55 ± 0.07	–64.40

a The results from the three replications conducted with AMBER are presented in Rep #1, Rep #2, and Rep #3.

**Figure 3:**
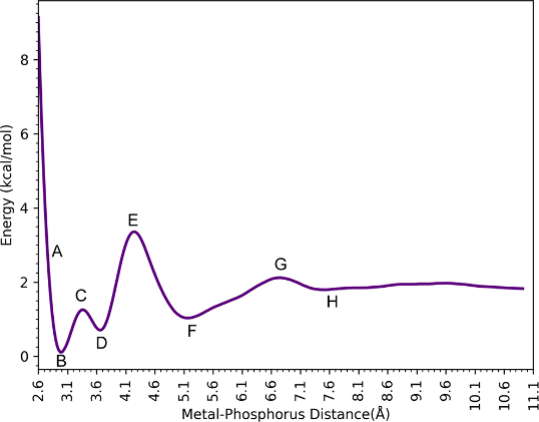
Binding free energy profiles of H_2_PO_4_^–^ interacting with Ca^2+^.

**Figure 4:**
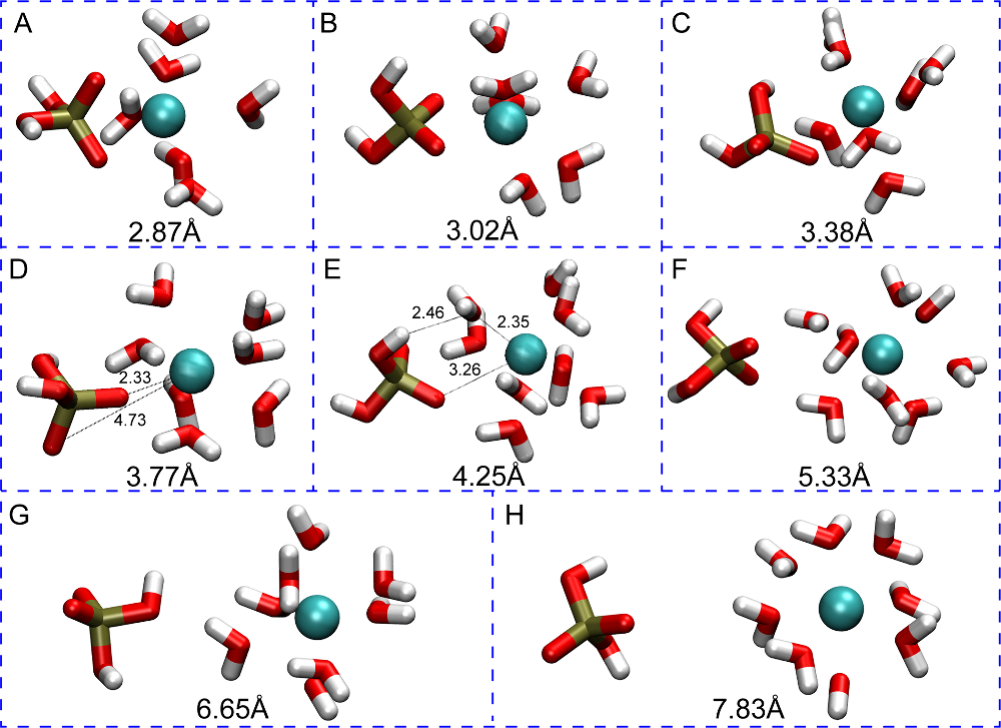
Representative snapshots from the simulation of H_2_PO_4_^–^ interacting with Ca^2+^. The large number in each panel shows the distance between the center of mass (COM) of the phosphorus atom and the metal ion. The smaller numbers in each panel show the distance between the atoms of interest in angstroms.

Figure 3 shows the free energy profiles along with the reaction coordinate defined by the phosphorus atom of the H_2_PO_4_^–^ interacting with Ca^2+^, with representative snapshots of H_2_PO_4_^–^ interacting with Ca^2+^ via the 12–6–4 LJ model. These profiles have a rich set of transition states and minima, which are shown in Figure 4 (using the labels from Figure 3) and described in more detail below for the H_2_PO_4_^–^ - Ca^2+^ complex. The remaining plots, namely HPO_4_^2–^ - Ca^2+^ and PO_4_^3–^ -Ca and their snapshots are given in the SI (see Figures S1–S4).

At 2.87 Å (Point A in Figure 3), the Ca^2+^ ion in the H_2_PO_4_^–^ - Ca^2+^complex has a coordination number of 8 with six water molecules and two oxygen atoms from the phosphate species. In this case, the phosphate has a bidentate interaction with Ca^2+^. The global minimum (B) is located at 3.02 Å (Ca–P), and Ca^2+^ is surrounded by 6 water molecules with Ca–O distances ranging from 2.47 to 2.56, and phosphate preserves its bidentate interaction. For the first saddle point (TS1) (C), one of the deprotonated oxygens moves away, while another water molecule approaches the metal center. Then, at 3.77 Å (Ca–P) (D), the H_2_PO_4_^–^ molecule rotates, and one of the deprotonated oxygens becomes closer to Ca^2+^ (2.33 Å) while the other moves away (4.73 Å). For the second transition state structure (E), the distance between the bound oxygen and Ca^2+^ is 3.26 Å and H_2_PO_4_^–^ rotates as the deprotonated oxygen faces bulk water and the hydrogens on the protonated oxygens interact with Ca^2+^ indirectly through water molecules (2.46 Å). As a result, the H_2_PO_4_^–^ molecule is in the second solvation shell of Ca^2+^ (F). At a 6.65 Å Ca–P distance (G), the third transition state structure has a low barrier and represents the shifting of the H_2_PO_4_^–^ molecule to the third solvation shell of Ca^2+^(H).

The initial structure of the HPO_4_^2–^ - Ca^2+^ interaction (Figures S1 and S2) is slightly different from that of H_2_PO_4_^–^. It starts with a tridentate binding of deprotonated oxygens, with approximate distances of 2.49 Å, 2.52 Å, 2.64 Å while the coordination number of Ca^2+^ is 9 (point A in Figure S1). However, in the global minimum (B), two oxygen atoms move away from 2.49 to2.61 Å and from 2.64 to2.77 Å. Meanwhile, another deprotonated oxygen remains at a constant distance from Ca^2+^ (2.50 Å), and the coordination number of Ca^2+^ remains at 9. At 3.29 Å Ca–P distance, the first transition state (tridentate to bidentate (C)) is located with a relatively high energy barrier (∼4 kcal/mol). At the 3.62 Å Ca–P distance (D), one water molecule approaches Ca^2+^ while one of the deprotonated bidentate oxygens moves away, leading to indirect binding through the water molecule (at 3.94 Å Ca–P distance) and Ca^2+^ keeps its coordination number at 8. In the second transition state structure (E), the deprotonated oxygen is moving away from the metal ion, with a distance of 2.94 Å between them, while the metal ion interacts with 8 water molecules through hydrogen bonds. Consequently, HPO_4_^2–^ shifts to the second solvation shell, and it interacts with Ca^2+^ in a bidentate manner through the water molecules (F) (2.69 and 1.72 Å). At 6.50 Å Ca–P distance, the molecule rotates, and the hydrogens on the protonated oxygens interact with the calcium ion through the first solvation shell water molecules. However, at 7.70 Å Ca–P distance (G), the molecule rotates while both of the deprotonated oxygens interact with the water molecules in the second shell. Afterward, the molecule fully disassociates (I).

On the other hand, the dissociation of PO_4_^3–^ from Ca^2+^ (Figures S3 and S4) has an energetically less favorable free energy pathway, while the trend of the dissociation is similar to the other phosphate derivatives. The dissociation starts with a global minimum structure (point B in Figure S3) in which PO_4_^3–^ has a tridentate interaction with Ca^2+^ (2.39 Å, 2.43 Å, 2.74 Å). A small shoulder (around 3.31 Å Ca–P distance) represents a Ca---O distance of 2.47 Å, and it leads to the bidentate interaction of PO_4_^3–^ with Ca^2+^ (C). Afterward, the distance of one of the oxygens stretches to 3.73 Å and this results in monodentate binding (D). At 4.02 Å Ca–P distance (E), the O–Ca^2+^ distance is 2.69 Å, and the binding mode is monodentate. At 4.81 Å Ca–P distance (F), there is a minimum where PO_4_^3–^ is in an indirect bidentate interaction with the metal ion mediated by water molecules. For a Ca–P distance between 5.00 Å to 7.47 Å (G and H), several shoulders that represent the dissociation of PO_4_^3–^ from the second solvation shell of Ca^2+^. After 7.47 Å (H), PO_4_^3–^ dissociates completely (I).

**Figure 5:**
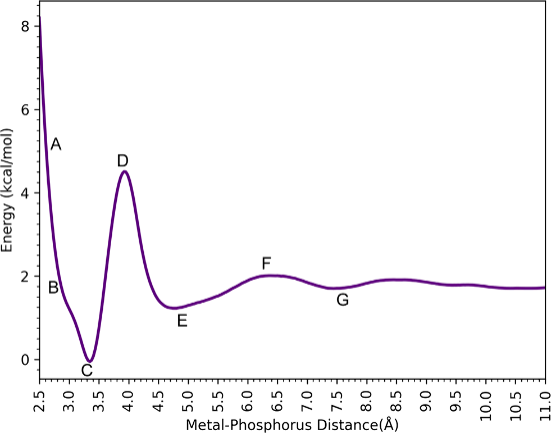
Binding free energy profiles of H_2_PO_4_^–^ interacting with Mg^2+^.

**Figure 6:**
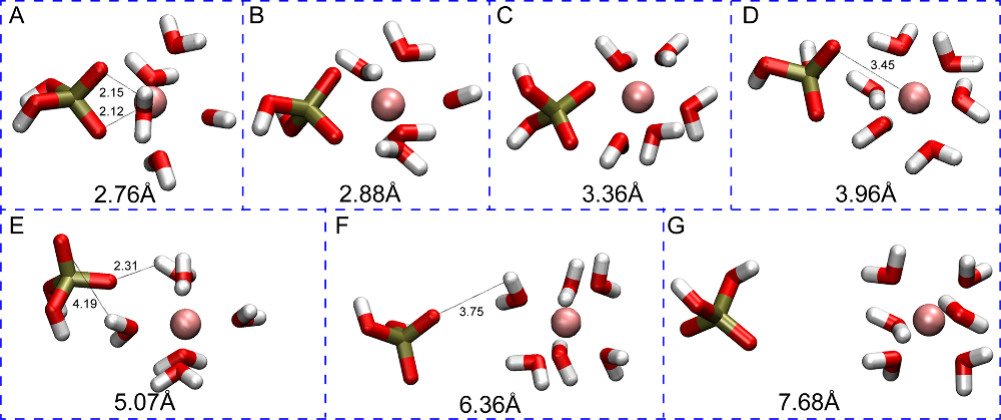
Representative snapshots of H_2_PO_4_^–^ interacting with Mg^2+^. In each panel, the smaller numbers show the distance between the atoms in angstroms, while the large number shows the distance between the COM of the phosphorus atom and Mg^2+^.

Figure 5 shows the free energy profiles along with the reaction coordinate defined by the phosphorus atom of H_2_PO_4_^–^ interacting with Mg^2+^, with representative snapshots of H_2_PO_4_^–^ interacting with Mg^2+^ via the 12–6–4 LJ model (Figure 6). The profile of HPO_4_^2–^ with Mg^2+^ is reported in SI together with representative snapshots taken from the simulation (see Figures S5 and S6).

The H_2_PO_4_^–^ - Mg^2+^ complex exhibits a different pattern than the other complexes. The initial structure (point A in Figure 5) of this system is the interaction of deprotonated oxygens with the Mg^2+^ ion at around 2.15 and 2.12 Å. The coordination number of Mg^2+^ is 7, surrounded by 5 water molecules. First, a small shoulder (B) is observed, corresponding to a structure in which deprotonated oxygens are elongated to 2.31 and 2.18 Å. This structure (C) results in a monodentate binding of H_2_PO_4_^–^ and the coordination number of Mg^2+^ ion is 6. The first transition state from the global minimum occurs at 3.96 Å (D), in which another water molecule approaches the Mg^2+^, resulting in a seven coordinated first shell. Moreover, the deprotonated oxygen and Mg^2+^ distance is elongated to 3.45 Å. At around 5.07 Å (E) the H_2_PO_4_^–^ is located at the second shell of the cation and the binding is changed to an indirect manner mediated by water molecules. The distances of deprotonated oxygens of the phosphate to the hydrogen atoms of the first shell water molecule are calculated as 2.31 and 4.19 Å, which results in a water mediated monodentate interaction. As the distance Mg–P elongates to 6.36 Å (F), a small transition state is observed. The distance between the deprotonated oxygens of H_2_PO_4_^–^ and the hydrogens of the first shell water molecule is increased from 2.31 to3.75 Å. After 7.00 Å (G), small shoulders represent the complete dissociation of H_2_PO_4_^–^ from the second solvation shell.

In comparison, HPO_4_^2–^ has a starting structure with tridentate interaction with Mg^2+^ (2.15 Å, 2.85 and 2.19 Å) (point A in Figure S5). The global minimum at 2.74 Å Mg–P distance (B), features one of the deprotonated oxygen moves to 3.59 Å resulting in bidentate binding mode (Figures S6 and S7). At around a 3.67 Å Mg–P distance (C), one of the deprotonated oxygens is positioned at a distance of 2.18 Å, leading to a monodentate binding mode. At a distance of 4.31 Å, the deprotonated oxygens indirectly interact with Mg^2+^ through the water molecules in the first hydration shell (D). Subsequently, these water-mediated interactions extend to a Mg–P distance of 6.28 Å(E). After this distance, HPO_4_^2–^ shifts to the third solvation shell and dissociates from the metal ion (F and G).

### Comparative Analysis between the 12-6 LJ AND 12-6-4 LJ Parameter Sets

**Figure 7:**
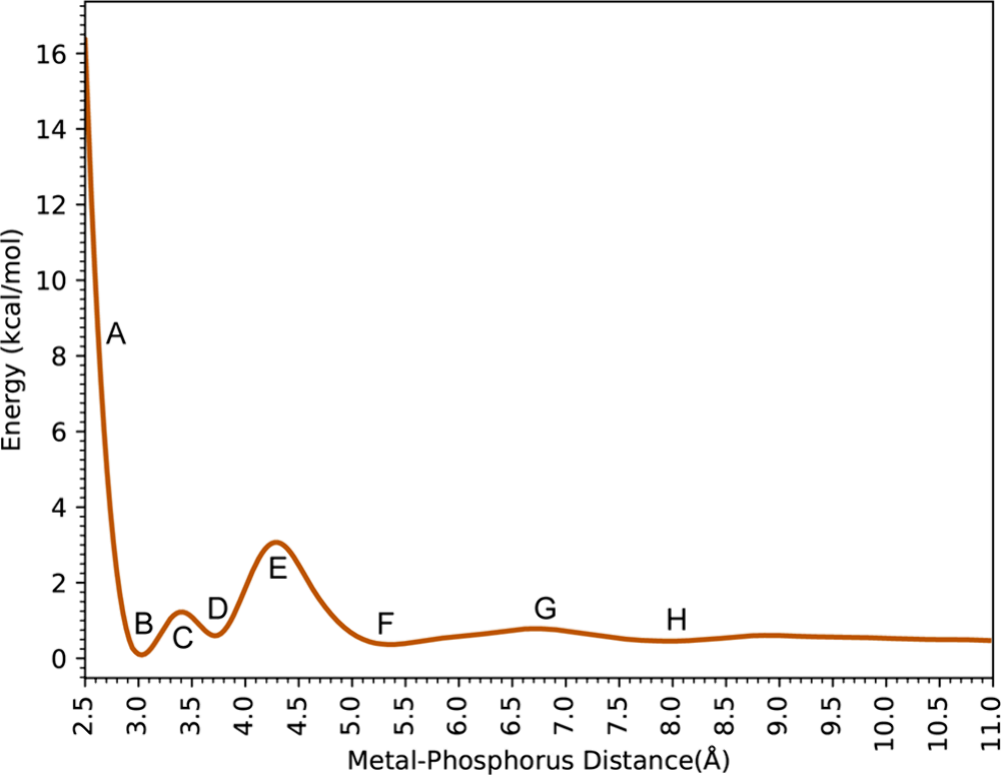
Free energy profiles showing the binding interaction between H_2_PO_4_^–^ and Ca^2+^.

Comparing Figure 7 and Figure 3, the difference shows that without the 12–6–4 LJ model, the 12–6 energy profile differs significantly in the energy of local minima and transition states but only differs slightly in the distances.

**Figure 8:**
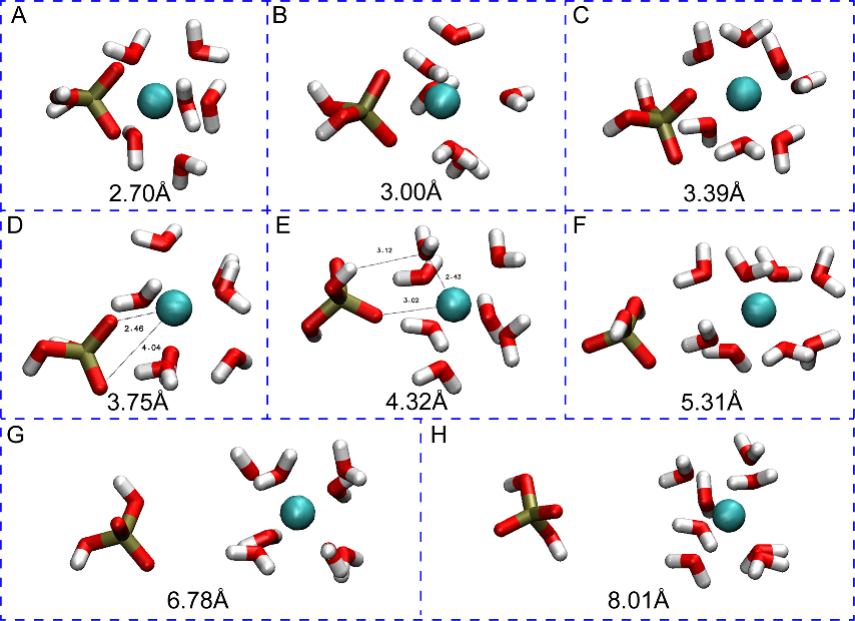
Representative snapshots from the simulation of H_2_PO_4_^–^ interacting with Ca^2+^. The smaller numbers in each panel show the distance between the atoms of interest in angstroms, while the large number indicates the distance between the COM of the phosphorus atom and Ca^2+^.

All the structures of the local minima and maxima are shown in Figure 8, whose labels are associated with the alphabetic tags in Figure 7 and described in more detail below.

At 2.70 Å (Point A), the Ca^2+^ ion in the 12–6 model has a coordination number of 8 with six water molecules and two oxygen atoms from the phosphate species, very similar to the energy profile calculated by the 12–6–4 model. The global minimum (B) located at 3.00 Å (Ca–P) and the first transition state at 3.39 Å (C) also show similar structures compared to the 12–6–4 model, where the Ca^2+^ ion is first surrounded by 6 water molecules plus the bidentate phosphate, then one of the deprotonated oxygens is replaced by a water molecule. At 3.75 Å (Ca–P) (D), the H_2_PO_4_^–^ repositions itself to have the still-bound deprotonated oxygen pointing at the Ca^2+^, yet the distance is much longer (2.46 Å) compared to the Ca–O distance in the 12–6–4 model (2.33 Å) likely due to a weaker 12–6 interaction between Ca^2+^ and O^–^.

Since the phosphate rotates less, the other oxygen only slightly moves away (4.04 Å) but not as far as the 12–6–4 model (4.73 Å). At the transition state (E, 4.32 Å), where the 12–6 model has much lower activation energy compared to the 12–6–4 model, the distance between the bound oxygen and Ca^2+^ is 3.02 Å, which is lower than the 3.26 Å in the 12–6–4 model. This proves that the 12–6–4 model maintains a stronger interaction between Ca^2+^ and O^–^. Because only by this stronger interaction, the 12–6–4 model will allow the ligand exchange to happen at a longer distance than the 12–6 model as the phosphate leaves Ca^2+^, still, the H_2_PO_4_^–^ maintains its interaction with Ca^2+^ indirectly through water molecules (3.12 Å for O^–^ - WAT and 2.43 Å for WAT - Ca^2+^) which is consistent between the 12–6 and 12–6–4 models. The rest parts of the energy profile, where the H_2_PO_4_^–^ molecule is in the second solvation shell of Ca^2+^ (F, 5.31 Å), experiencing ligand exchange between the second and third solvation shells (G, 6.78 Å) and in the third solvation shell of Ca^2+^(H, 8.01 Å) are very similar to the 12–6–4 model.

The initial structure of the HPO_4_^2–^ - Ca^2+^ interaction using the 12–6 model is also similar to the 12–6–4 model-generated structure (Figures S7 and S8). The phosphate group also forms a tridentate chelating to the Ca^2+^ ion. However, in the global minimum (B, 2.77 Å), two oxygen atoms move away at distances (2.57 and 2.83 Å), which are very different from the 12–6–4 model (2.61 and 2.77 Å). At the same time, the third deprotonated oxygen keeps almost the same distance to Ca^2+^ (from 2.40 to2.42 Å).

When the Ca–P distance reaches 3.61 Å, the first saddle point (C) happens with a significantly higher energy increase (∼8 kcal/mol) compared to the 12–6–4 model (∼4 kcal/mol). This means for the 12–6 system, Ca^2+^ has a coordination number change from 9 to 8, where the 4.03 Å oxygen is driving phosphate rotation and dissociation simultaneously. This adds a higher rotational penalty on the phosphate molecule compared to the 12–6–4 model (see points C and D in Figure S2, where rotation happens before dissociation, not at the same time).

After this rapid “rotating and leaving” state, the monodentate conformation (D, 4.30 Å) stays mostly the same shape as the 12–6–4 model (2.97 Å Ca^2+^ - O^–^ distance) yet at a much longer distance (3.45 Å) due to weaker C_4_ interactions between Ca^2+^ and O^–^. Then, at the Ca–P distance of 4.90 Å (E), HPO_4_^2–^ shifts to the second solvation shell and forms a phosphate-water-Ca^2+^ hydrogen bonding network that has an 8-member hexagonal ring (OP···HW-OW is 180°) structure. The subsequent second-to-third transition state at 6.73 Å Ca–P distance (F), as well as the third solvation shell local minima (G, 7.57 Å), are both similar to the 12–6–4 model in shape, but have longer distance due to weaker C_4_ intermolecular forces.

Similarly, in the PO_4_^3–^ -Ca^2+^ system (Figures S9 and S10), we also observed significant differences that happen during the first-second solvation shell transition between 12 and 6 and 12–6–4 models for H_2_PO_4_^–^ - Ca^2+^and HPO_4_^2–^ - Ca^2+^ At the beginning, the trend of dissociation is similar between the 12–6 and 12–6–4 models. The global minimum (Point A, 2.60 Å) is very similar compared to Figure S4(point B). But when the transition (B, 3.59 Å) happens, it shows a stronger phosphate rotation (3.90 and 2.42 Å for the two deprotonated oxygens) compared to the 12–6–4 model (Point C in Figure S4, 2.98 and 2.47 Å for the two deprotonated oxygens). This, again, causes a huge energy penalty, so from conformation A to B, the energy increase is ∼17 kcal/mol, yet for the 12–6–4 system, the energy increase is only ∼6 kcal/mol.

After the rotation and dissociation, the coordinating oxygen has a distance of 3.37 Å to the Ca^2+^ (C, 4.31 Å Ca–P distance) and forms a monodentate binding. This, compared to Figure S4 (Point E, 2.69 Å Ca2+-O- distance), is very energetically unfavored, so the significant drop from E to F in Figure S3 is not observed here from C to D in the 12–6 model PMF. From this point on, the second-to-third transition state (E, 6.71 Å, compared to point G in Figure S4) and third solvation shell minimum (F, 7.48 Å, compared to point H in Figure S4) are very similar.

**Figure 9:**
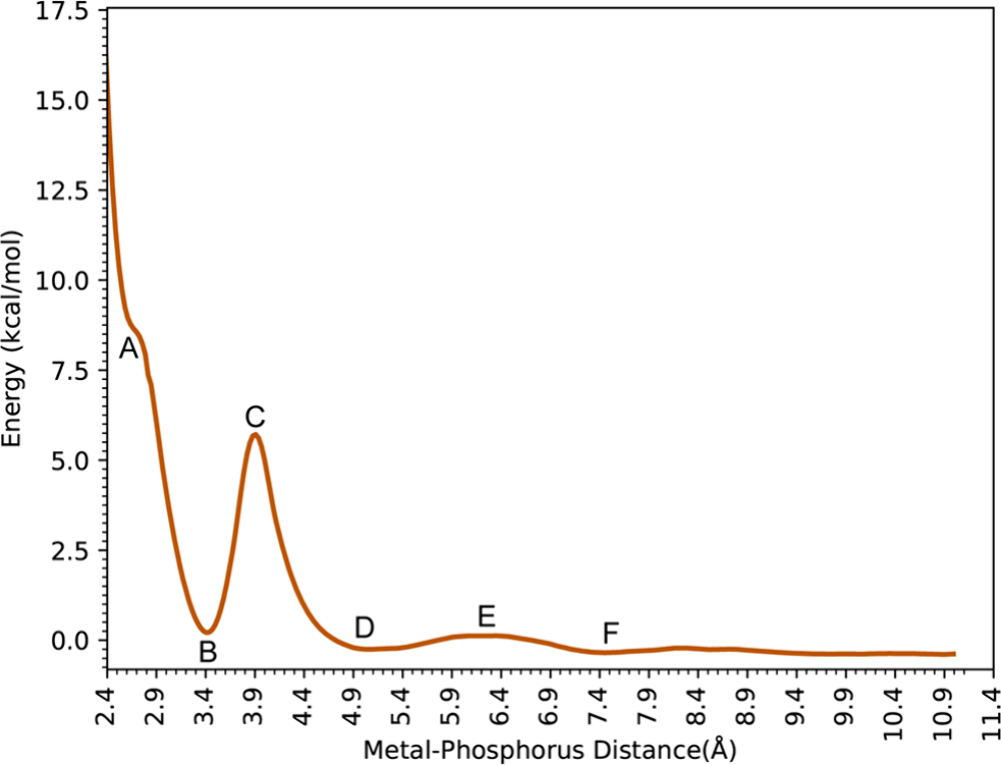
Binding free energy profiles of H_2_PO_4_^–^ interacting with Mg^2+^.

**Figure 10:**
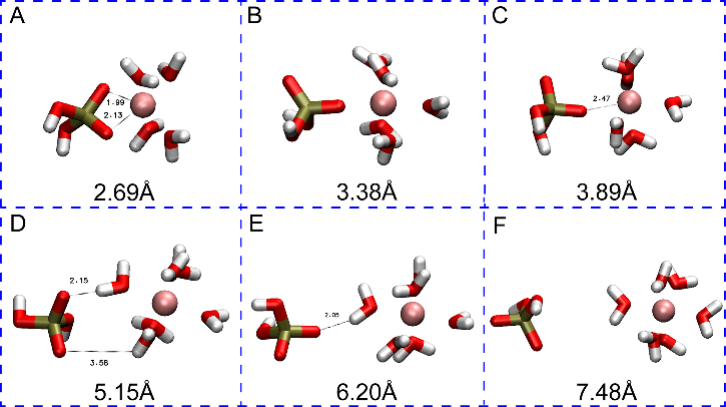
Representative snapshots from the simulation of H_2_PO_4_^–^ interacting with Mg^2+^. In each panel, the large number indicates the distance between the COM of the phosphorus atom and the metal ion, while the smaller numbers indicate the distances between the atoms of interest. All distances are measured in angstroms.

Figures 9 and 10 show the free energy profiles along with the reaction coordinate defined by the phosphorus atom of H_2_PO_4_^–^ interacting with Mg^2+^, with representative snapshots of H_2_PO_4_^–^ interacting with Mg^2+^ via the 12–6 model and can be compared with Figures 5 and 6 side-by-side.

By comparing the two different LJ models for the H_2_PO_4_^–^ - Mg^2+^ complex, the major difference happens at the formation of the second solvation shell hydrogen bond network (D, 5.15 Å, compared to Figure 6E). At a PMF plateau of 2.69 Å Mg–P distance, two deprotonated oxygens chelating to the Mg^2+^ ion at around 1.99 and 2.13 Å, forming a 7 coordinate first solvation shell with five other water molecules. Then, the local minimum (B) forms a monodentate binding of H_2_PO_4_^–^ and the coordination number of Mg^2+^ ion reduces to 6.

The first transition state from the global minimum occurs at 3.89 Å (C, compared to Figure 6D), where another water molecule approaches the Mg^2+^ and tries to replace the monodentate. Note here the difference between the 12–6 and 12–6–4 models appears, where in the 12–6 model, even though the Mg–P distance is longer, the Mg–O distance is still shorter than the 12–6–4 model (Figure 6D dashed line). This indicates that the water will take more effort to replace the phosphate in this 12–6 model, compared to the 12–6–4 model, causing a higher energy barrier (∼6 kcal/mol, whereas in 12–6–4 it is ∼4 kcal/mol).

Later, when the phosphate moves to the second solvation shell, the deprotonated oxygen and Mg^2+^ distance is elongated to 5.15 Å (D, compared to Figure 6E), and the hydrogen bond network is sturdier than the 12–6–4 model (2.15 and 3.58 Å, compared to 2.31 and 4.19 Å in Figure 6E). This results in a ∼ 6 kcal/mol energy drop from C to D, compared to the ∼3 kcal/mol drop from D to E in Figure 6, causing the two PMFs to differ between the 12–6 and 12–6–4 models. As the Mg-p distance elongates to 6.20 Å (E), a small transition state appears to break a 2.05 Å interaction between the deprotonated oxygens of H_2_PO_4_^–^ and the hydrogens of the first shell water. At 7.48 Å (F), the local minimum indicates the phosphates’ residence at the third solvation shell.

In comparison, HPO_4_^2–^ - Mg^2+^ has the most significant difference between the 12–6 model-generated energy profile and the 12–6–4 model-generated one (Figures S11 and S12), and the difference is mostly located at the transition between conformation C and D (if comparing Figures S12 and S6). The PMF with the 12–6 model starts with a structure of HPO_4_^2–^ identically chelating with Mg^2+^ (1.88 Å, 1.92 Å, and 1.95 Å) (point A in Figure S12).

The global minimum at 2.30 Å Mg–P distance (B) still maintains a tridentate conformation, while in the 12–6–4 model, one of the deprotonated oxygen moves to 3.59 Å resulting in bidentate conformation (Point B in Figure S6). This difference did not cause the PMFs to differ between the 12–6 and 12–6–4 models because it eventually leads to the same monodentate conformation at a turning point on the PMF (C, 3.20 Å, similar to point C in Figure S6). However, at the distance of 3.95 Å, the ligand-water exchange happens, which is at a distance much shorter than the 12–6–4 model’s ligand-water exchange distance (4.30 Å, see point D in Figure S6) due to the C_4_-less interaction between Mg^2+^ and O^–^.

Note here the C_4_ is repulsive, so the 12–6–4 model will have a longer Mg^2+^-O^–^ distance when the water exchange happens. This makes the end conformation (from point C to D in Figure S6) more energetically favored. However, in the 12–6 model here, conformation D is less energetically favored compared to C due to the steric hindrance between water and the phosphate, which is not fully leaving the first solvation shell yet. Then, when the Mg–P distance increases to 4.31 Å (E), the energy is slightly favored, so there is a slight energy drop from D to E. After this distance, HPO_4_^2–^ gradually shifts to the transition state (F, 6.20 Å) between the second and the third solvation shells. Eventually, the PMF will have its final local minimum, which is associated with the third solvation shell (G, 6.98 Å).

To further validate our optimized parameters, we compared the distances between the metal ion and the phosphate group in the crystal structures of DNA-metal ions complexes with the H_2_PO_4_^–^ derivative parameters used in the current study. Since in the structure of DNA, only two oxygen atoms of the phosphate group are free while the other two are involved in phosphodiester bonds, we selected H_2_PO_4_^–^ as the exemplar for the phosphate group in the DNA backbone. We performed MD simulations on five crystal structures of DNA-magnesium complexes (PDB IDs 1PUY and 3DNB) and DNA-calcium complexes (PDB IDs 1SK5, 2GW0, and 1ZF6). Afterward, we measured the distances between the metal ions and various atoms of the phosphate group (see Table S3). Our analysis revealed that when applying our optimized 12–6–4 LJ parameters, the measured distances matched those in the corresponding crystal structures better than those using 12–6.

However, in systems using the default 12–6 LJ parameters, the metal ions showed reduced interactions with the corresponding phosphate groups, as indicated in Table S3. For example, in the system with the 1SK5 crystal structure, Ca^2+^ dissociated from the phosphate group (Figure S13). As a result, an additional water molecule interacted with the metal ion to compensate for the phosphate oxygen, in contrast to both experimental data and simulations using 12–6–4 LJ parameters (Figure S13). Moreover, with the 12–6 LJ parameters, we observed a conformational change in one of the DNA structures (PDB ID 1ZF6) when the metal ion coordinating with deoxycytidine (DC)2 and DC12 lost its interactions with DC2 (Figure S14). On the other hand, the conformation obtained with 12–6–4 LJ parameters closely matched the original crystal structure. It is evident that the systems with 12–6 LJ parameters displayed significant deviations from experimental data, as shown in Figures S13 and S14. However, upon applying our optimized parameters, the distances between phosphate atoms and metal ions and their interactions are adjusted and align with the experimental data obtained from crystal structures.

## Conclusion

In this study, we have improved the 12–6–4 LJ non bonded model for H_2_PO_4_^–^, HPO_4_^2–^, and PO_4_^3–^ interacting with Ca^2+^ and Mg^2+^. The polarizability values are parametrized against the experimental hydration free energy values for each phosphate derivative and the experimental absolute binding free energy values for metal cation–phosphate pairs. We observed significant differences in the energy of transition states for both Ca^2+^ and Mg^2+^ complexes when using 12–6 LJ and 12–6–4 LJ potentials. The optimized 12–6–4 LJ potential was able to reproduce experimental values using PMF calculations. One of the key findings of the present study is the significant alteration in both the energy of the transition states and the location of the local minimum (i.e., the distance between the phosphorus atom and magnesium) when applying our optimized parameter sets in, for example, the HPO_4_^2–^ - Mg^2+^ complex. This highlights the importance of employing optimized 12–6–4 LJ parameter sets when investigating the influence of Ca^2+^ and Mg^2+^ on biomacromolecules such as DNA and RNA as the phosphate groups form the foundational structure of these macromolecules. The newly established parameters in this study will facilitate the simultaneous acquisition of thermodynamic and structural information, which can be used in a range of biomolecular simulations.

## Supporting Information Available

Hydration free energy calculations of phosphate derivatives at QM level, binding free energy values, binding free energy profiles, representative snapshots from the simulation of HPO_4_^2–^ interacting with Ca^2+^, and additional supplemental data (PDF)
