# Supplementary material for: Binding of Phosphate Species to Ca2+ and Mg2+ in Aqueous Solution
Source: J Chem Theory Comput. 2024 May 8;20(10):4298–307. doi: 10.1021/acs.jctc.4c00218 (PMC11137831; doi:10.1021/acs.jctc.4c00218)
Supplement: Supplementary file 1 — ct4c00218_si_001.pdf [file ct4c00218_si_001.pdf]

Supporting Information

Binding of Phosphate Species to Ca^{2+} and Mg^{2+} in Aqueous Solution

Basak Koca Fındık[†¥], Majid Jafari[#¥], Lin Frank Song[§], Zhen Li[‡], Viktorya Aviyente[†], Kenneth M. Merz, Jr.[*‡#]

[†]Department of Chemistry, Bogazici University, Bebek 34342, Istanbul, Turkey

[#]Department of Biochemistry and Molecular Biology, Michigan State University, East Lansing, Michigan 48824, United States

[§]Biochemical and Biophysical Systems Group, Lawrence Livermore National Laboratory, Livermore, CA, 94550, United States

[‡]Department of Chemistry, Michigan State University, East Lansing, Michigan 48824, United States

**Corresponding Author:* Kenneth M. Merz

**Corresponding Author Email:* merz@chemistry.msu.edu

[¥] These authors contributed equally to this work.

Table S1. Hydration free energy calculations of phosphate derivatives at QM level (M06/6-311+G(d,p) and using thermodynamic cycle via one-step and two-step approaches. [a]Calculated via extrapolation of the values of other phosphate derivatives.

Molecules	Experimental (Marcus[1])	QM at M06/6-311+G(d,p)	One-Step TI	Two-Step TI
$H_2PO_4^-$	-111.06	-81.83	-111.12	-110.87
HPO_4^{2-}	-315.69[a]	-271.38	-315.36	-315.29
PO_4^{3-}	-660.85	-592.63	-661.14	-661.11

Table S2. Shows the binding free energy values (in kcal/mol) obtained using three different parameter sets (the default 12-6 LJ, the default 12-6-4 LJ, and the optimized 12-6-4 LJ).

System	Experimental values(kcal/mol)	Standard 12-6-4 (kcal/mol)	Standard 12-6 (kcal/mol)	Optimized 12-6-4(kcal/mol)
$H_2PO_4^-$ - Ca^{2+}	-1.9	0.11	-0.68	-1.96
HPO_4^{2-} - Ca^{2+}	-3.75	-10.06	-13.07	-3.54
PO_4^{3-} - Ca^{2+}	-8.8	-32.72	-32.51	-8.53
$H_2PO_4^-$ - Mg^{2+}	-1.73	7.04	0.61	-1.71
HPO_4^{2-} - Mg^{2+}	-3.86	-17.5	-35.47	-3.55

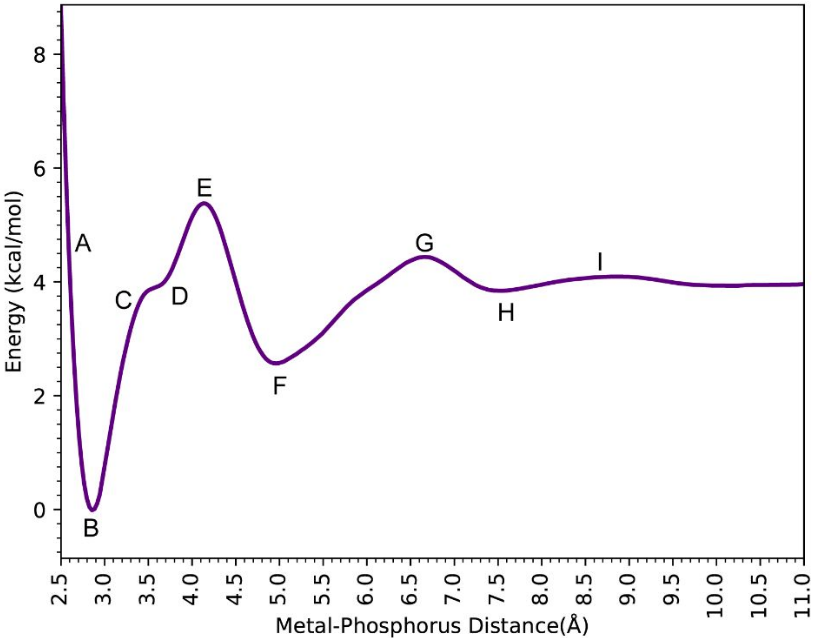

Figure S1. Binding free energy profiles of HPO_4^{2-} interacting with Ca^{2+}.

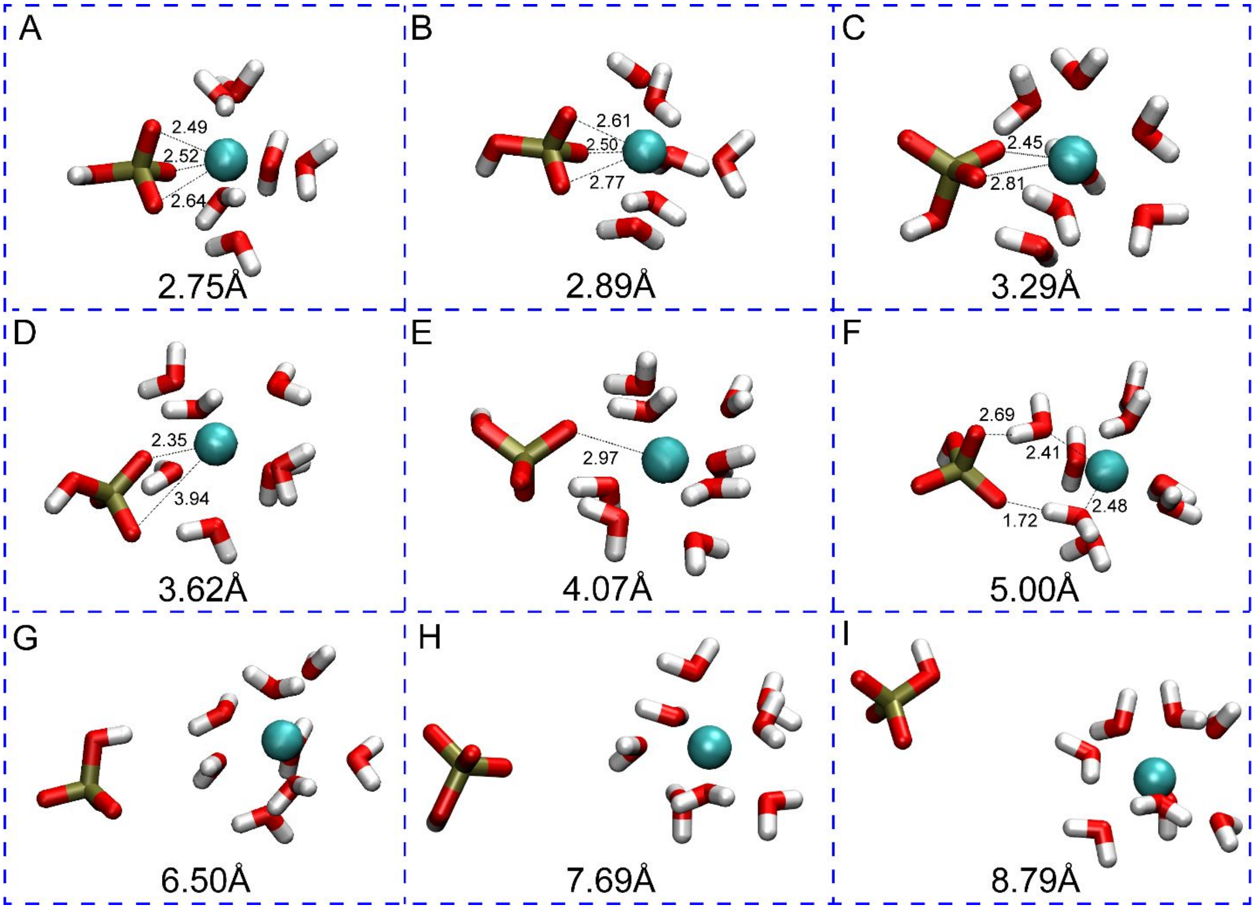

Figure S2. Representative snapshots from the simulation of HPO_4^{2-} interacting with Ca^{2+}. In each panel, the larger number indicates the distance between the center of mass (COM) of the phosphorus atom and the metal ion, while the smaller numbers indicate the distances between the atoms of interest. All distances are measured in angstroms.

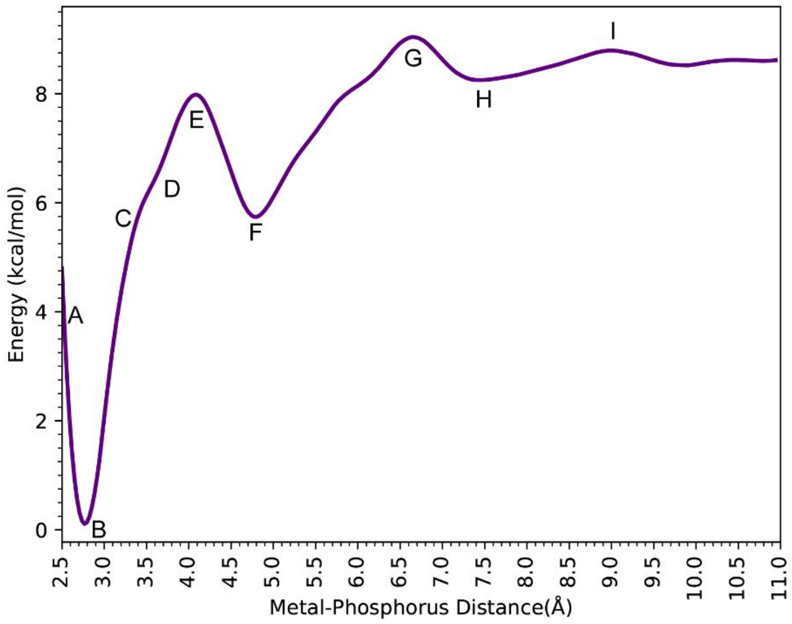

Figure S3. Binding free energy profiles of PO_4^{3-} interacting with Ca^{2+}.

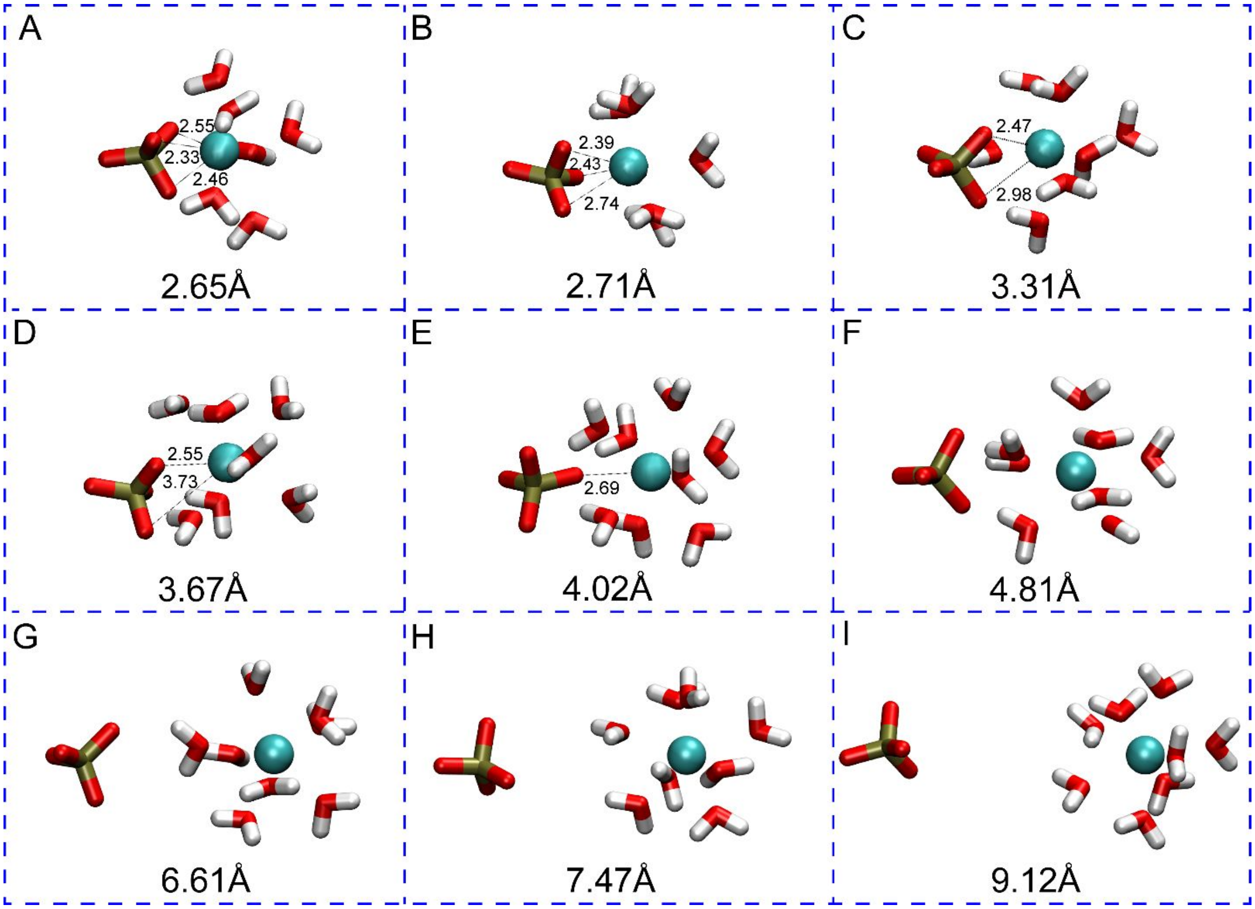

Figure S4. Representative snapshots from the simulation of PO_4^{3-} interacting with Ca^{2+}. The large number in each panel shows the distance between the COM of the phosphorus atom and the metal ion. The smaller numbers in each panel indicate the distance between the atoms of interest in angstroms.

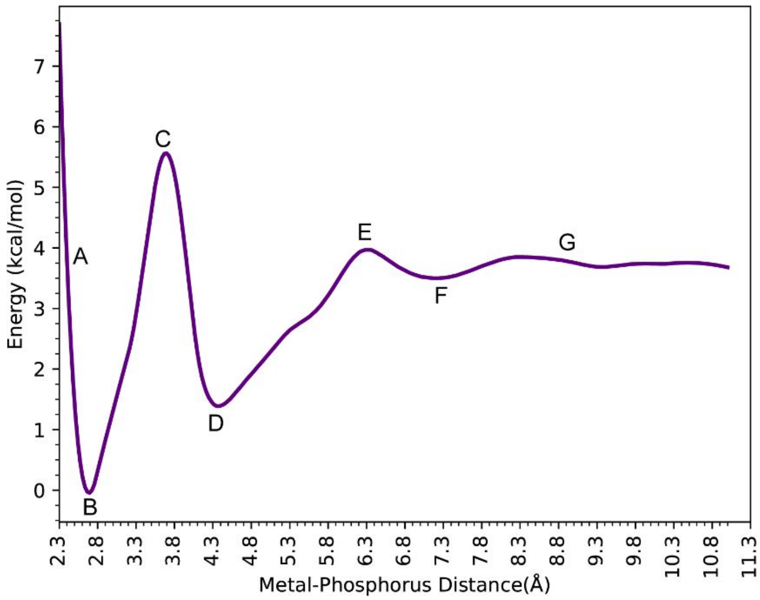

Figure S5. Binding free energy profiles of HPO_4^{2-} interacting with Mg^{2+}.

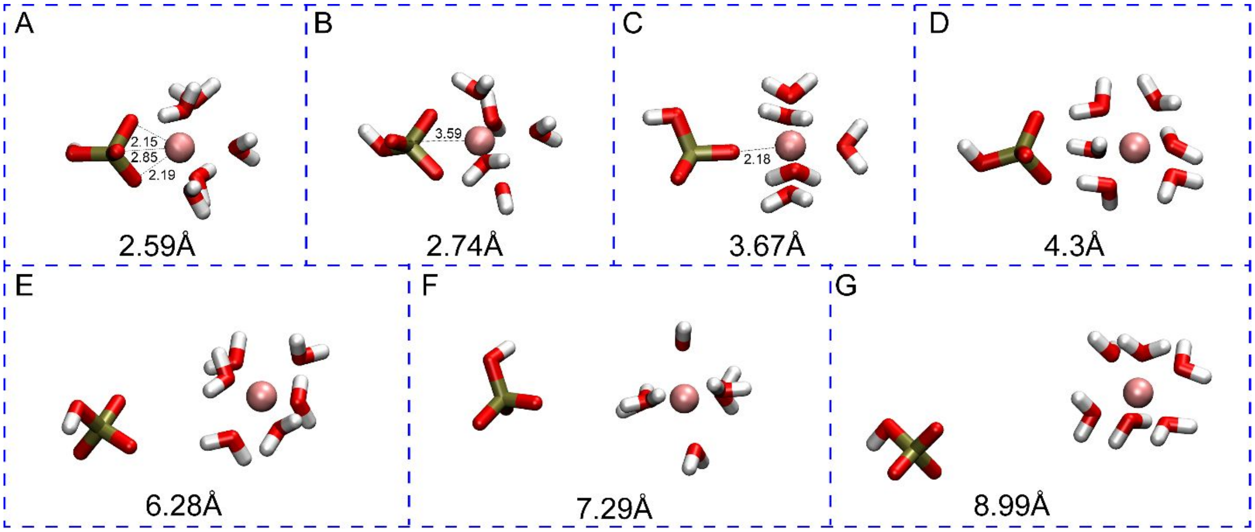

Figure S6. Representative snapshots from the simulation of HPO_4^{2-} interacting with Mg^{2+}. The large number in each panel indicates the distance between the COM of the phosphorus atom and the metal ion. The smaller numbers show the distance between the atoms of interest in angstroms.

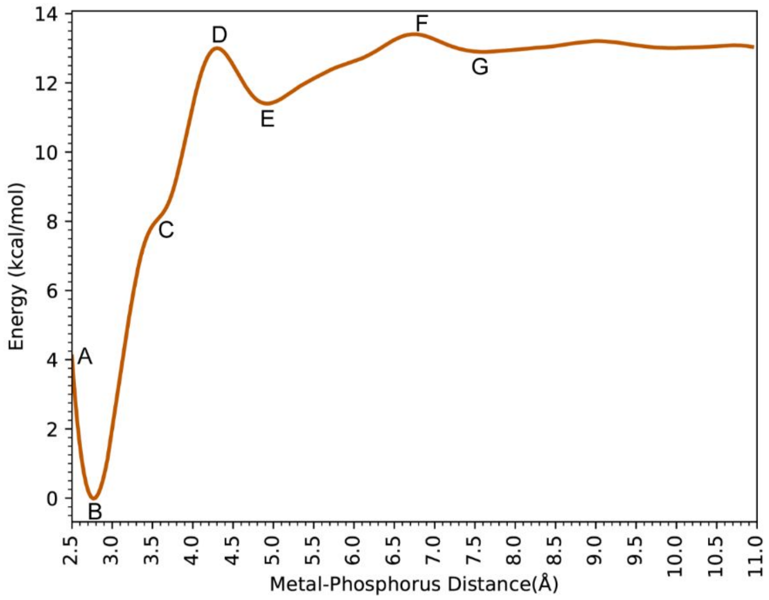

Figure S7. Free energy profiles showing the binding interaction between HPO_4^{2-} and Ca^{2+}.

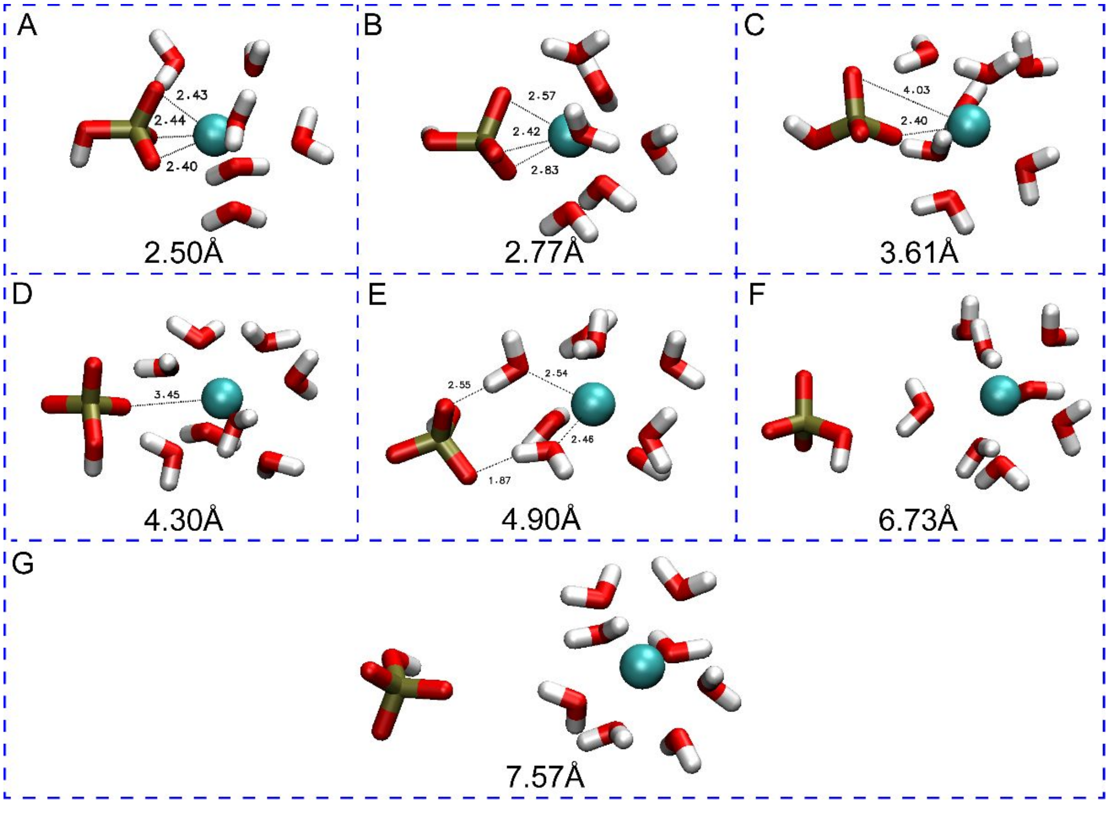

Figure S8. Represents the snapshots captured during the simulation depicting the interaction between HPO_4^{2-} and Ca^{2+} ions. The smaller numbers show the distance between the atoms of interest, while the larger number shows the distance between the COM of the phosphorus atom and Ca^{2+}.

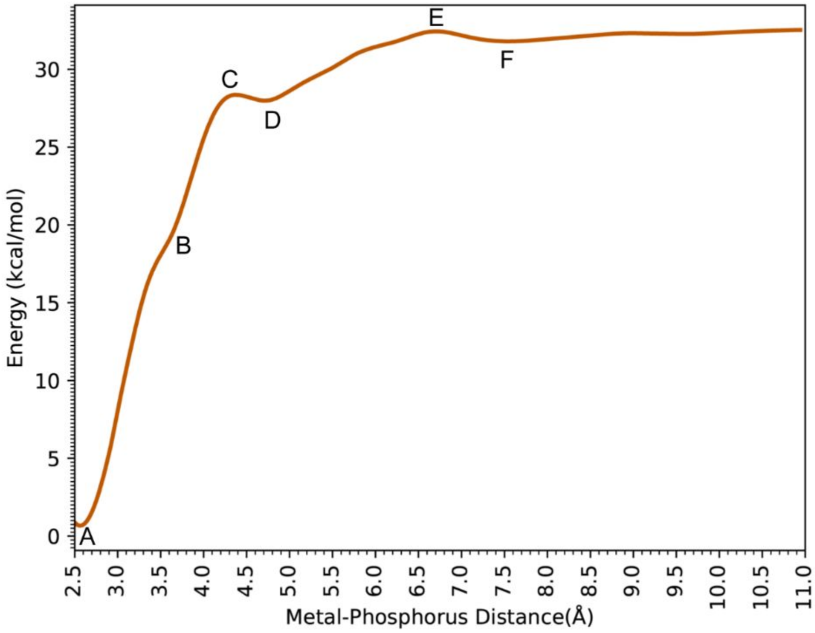

Figure S9. Binding free energy profiles of PO_4^{3-} interacting with Ca^{2+}.

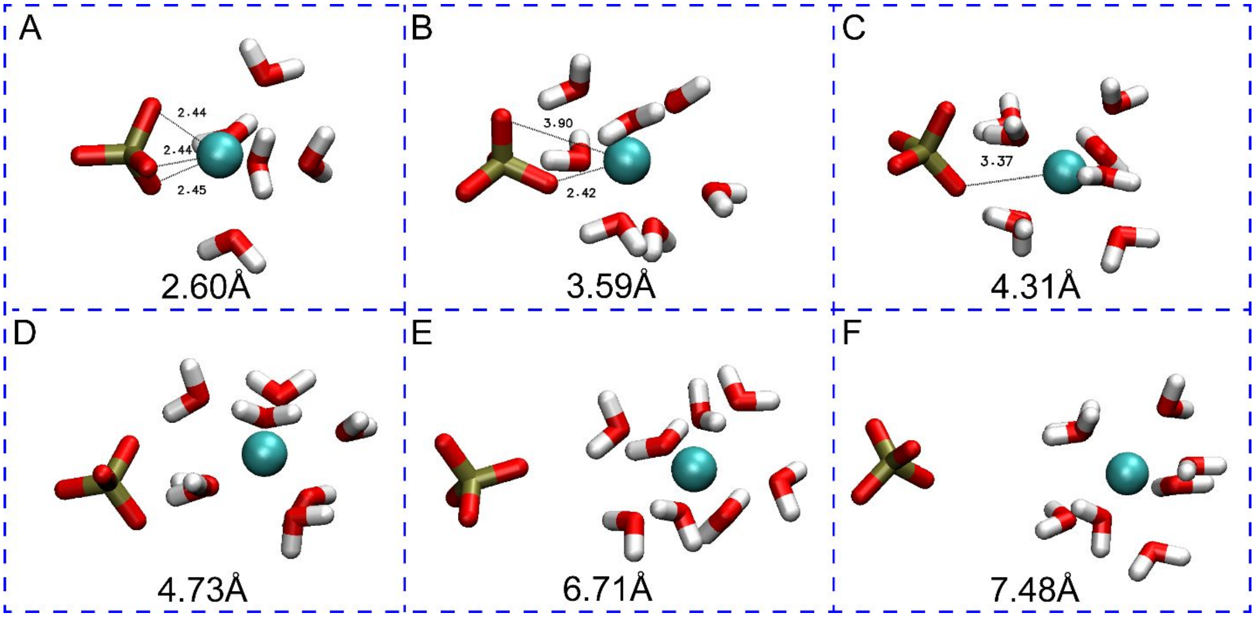

Figure S10. Representative snapshots from the simulation of PO_4^{3-} interacting with Ca^{2+}. The large number indicates the distance between the metal ion and the phosphorus atom where the snapshot is taken. The smaller numbers in each panel show the distance between the atoms of interest.

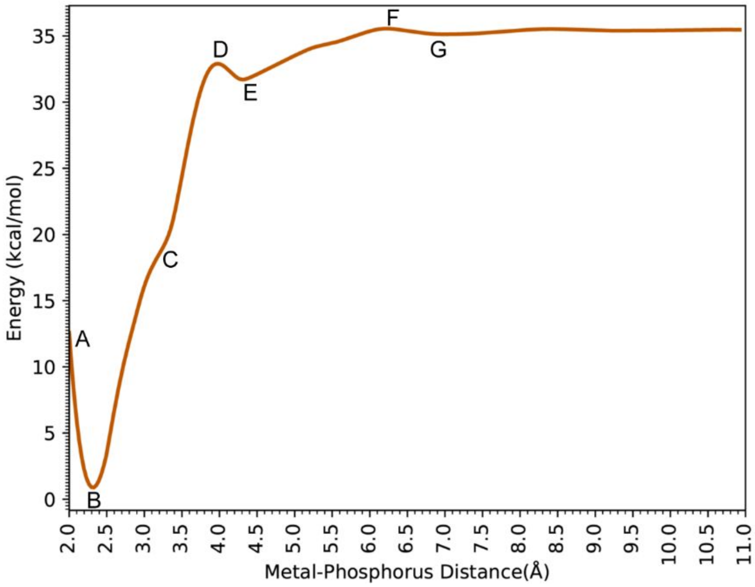

Figure S11. Binding free energy profiles of HPO_4^{2-} interacting with Mg^{2+}.

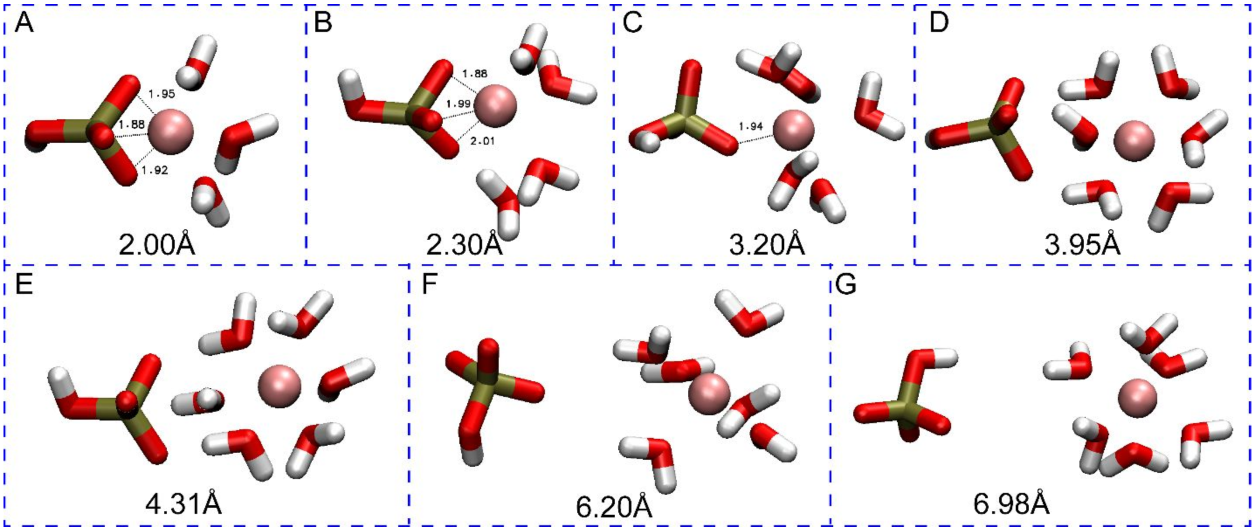

Figure S12. Representative snapshots from the simulation of HPO_4^{2-} interacting with Mg^{2+}. In each panel, the smaller numbers show the distance between the atoms in angstroms, while the large number shows the distance between the COM of the phosphorus atom and Mg^{2+}.

Table S3. Comparison of distances between Mg^{2+} and Ca^{2+} ions and various atoms of the phosphate group/water from DNA crystal structures using 12-6 LJ and 12-6-4 LJ parameters. OW and OP represent water oxygen and phosphate oxygen, respectively. Exp stands for the experimental distance. All distances are in angstroms. D represents the simulation systems, where the metal ion of interest dissociates from the phosphate group.

**PDB ID**	**Mg^{2+}-OW(Average)**			**OW-OP (Average)**			**Mg^{2+}-OP**		
	**Exp**	**12-6 LJ**	**12-6-4 LJ**	**Exp**	**12-6 LJ**	**12-6-4 LJ**	**Exp**	**12-6 LJ**	**12-6-4 LJ**
1PUY	~2.06	~2.16	~2.09	~2.95	**D**	~3.51	4.12	**D**	4.62
3DNB	~2.01	~2.13	~2.09	~2.85	~2.95	~2.72	2.01	1.97	1.95
**PDB ID**	**Ca^{2+}-OW(Average)**			**OW-OP (Average)**			**Ca^{2+}-OP**		
	**Exp**	**12-6 LJ**	**12-6-4 LJ**	**Exp**	**12-6 LJ**	**12-6-4 LJ**	**Exp**	**12-6 LJ**	**12-6-4 LJ**
1SK5	~2.42	~2.52	~2.49	~2.67	**D**	~2.93	4.04	**D**	2.23
2GW0	~2.39	~2.52	~2.48	~2.92	**D**	~2.78	4.29	**D**	2.35
1ZF6*	~2.79	~2.55	~2.46	~2.93	**D**, ~2.96	~2.72	2.41	**D**, 2.36	2.44

*The distances shown in this simulation are calculated between a single nucleotide (DC12) and calcium.

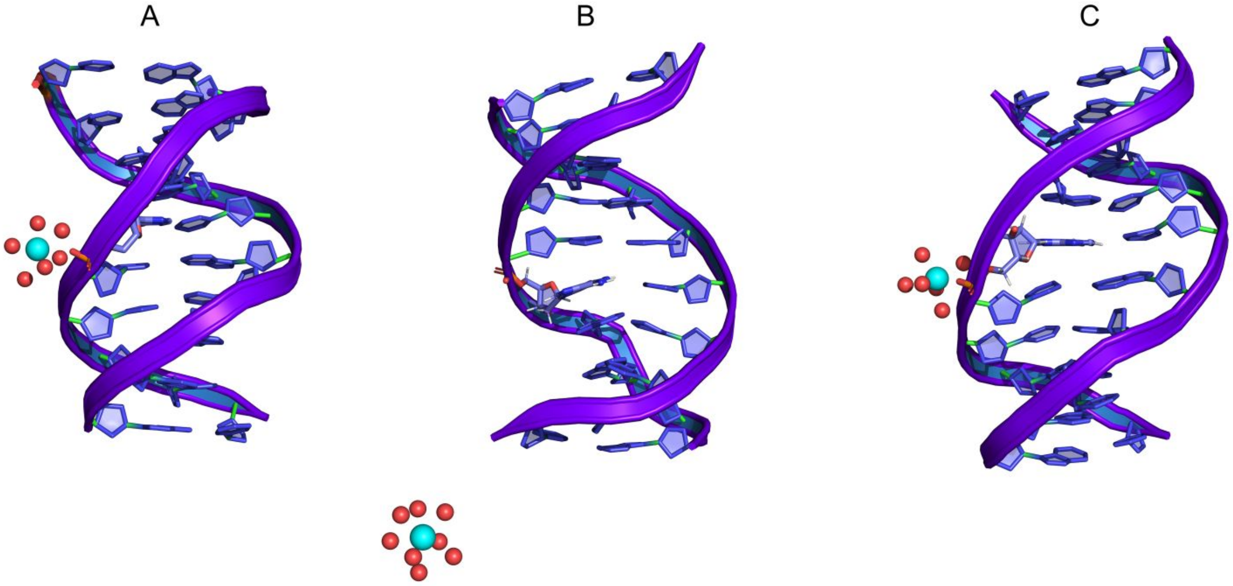

Figure S13. Represents the Ca^{2+}-phosphate interaction in simulation systems containing DNA. A) Depicts the calcium ion (cyan balls) surrounded by water molecules (red balls) in the crystal structure (PDB ID 1SK5). B) and C) represent the simulation results using 12-6 LJ and 12-6-4 LJ parameters, respectively.

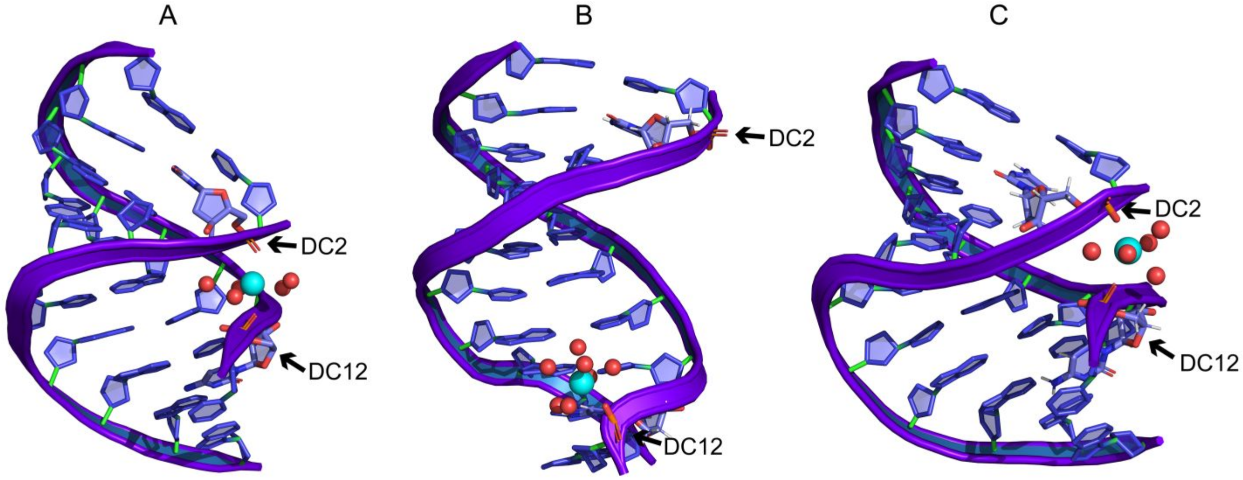

Figure S14. Represents Ca^{2+}-DNA interactions in the crystal structure (A, PDB ID 1ZF6). In B) and C), the DNA structure is shown alongside a calcium metal ion (cyan balls) surrounded by water molecules (red balls) in simulations with 12-6 LJ and 12-6-4 LJ parameters, respectively. DC stands for deoxycytidine.

Computational method for distance calculation

To prepare the necessary systems for distance calculations, two systems were established, each with 12-6 LJ and 12-6-4 LJ parameters for every metal ion. The crystal structures were obtained from the RCSB PDB databank, and the original water molecules surrounding the metal ions were retained. Afterward, the DNA structures were solvated in a box using the TIP3P water model, with K^+ and Cl^- ions used as counterions to neutralize the systems. Each system underwent energy minimization and equilibration phases, as described in detail in the method section. Then, a 10 ns production phase was performed on each system at 303 K. Following this, the last snapshot of each MD simulation system was extracted. Then the distances between various phosphate atoms/water molecules and the metal ion were computed using the cpptraj and VMD software packages.

Provided below are the charge values in the mol2 file used for the PMF calculation of the Ca-$H_2PO_4^-$ complex (see the last column):

1	P1	-0.0000	0.0640	0.0000	p5	1 LIG	0.514982
2	O1	-1.2750	-0.9770	-0.7280	oh	1 LIG	-0.514918
3	H1	-1.6160	-1.4980	-0.0000	ho	1 LIG	0.339387
4	O2	1.2780	-0.9740	0.7280	oh	1 LIG	-0.514918
5	H2	1.6190	-1.4950	0.0000	ho	1 LIG	0.339387
6	O3	0.7270	1.1040	-1.2760	o	1 LIG	-0.581961
7	O4	-0.7300	1.1020	1.2770	o	1 LIG	-0.581960

The charge values in the mol2 file applied in the PMF calculation for the HPO_4^{2-} - Ca^{2+} complex are presented below:

1	P	-0.397744	0.127669	-0.130675	p5	1 LIG	0.247036
2	O	0.430886	0.808971	-1.226631	o	1 LIG	-0.659702

3	O2	-0.102342	-1.362465	0.054924	o	1	LIG	-0.655223
4	O3	-1.891587	0.456934	-0.164000	o	1	LIG	-0.655242
5	O1	0.167904	0.858013	1.293383	oh	1	LIG	-0.580457
6	H	0.848883	1.490879	1.052999	ho	1	LIG	0.303588

The charge values in the mol2 file used in calculating the PMF for the PO_4^{3-} - Ca^{2+} complex are given below:

1	P1	0.0000	-0.3600	1.0000	p5	1	LIG	0.204931
2	O1	1.3060	0.1730	1.7540	o	1	LIG	-0.801235
3	O2	0.0000	-1.9600	1.0000	o	1	LIG	-0.801228
4	O3	0.0000	0.1730	-0.5080	o	1	LIG	-0.801233
5	O4	-1.3060	0.1730	1.7540	o	1	LIG	-0.801235

The PMF calculation for the $H_2PO_4^-$ - Mg^{2+} complex involves the following charge values as outlined in the mol2 file:

1	P1	-0.0000	0.0000	-0.1700	p5	1	LIG	0.483020
2	O1	0.9950	0.8280	0.9020	oh	1	LIG	-0.486815
3	H1	1.8110	0.3000	0.8950	ho	1	LIG	0.352842
4	O2	-0.9950	-0.8280	0.9020	oh	1	LIG	-0.486392
5	H2	-1.8110	-0.3000	0.8950	ho	1	LIG	0.353515
6	O3	-0.8870	1.0050	-0.8540	o	1	LIG	-0.499153
7	O4	0.8870	-1.0050	-0.8550	o	1	LIG	-0.505773

Presented below are the charge values in the mol2 file used for calculating the PMF of the Mg-HPO_4^{2-} complex:

1	P1	-0.1540	-0.0000	0.0680	p5	1	LIG	0.378522
2	O1	1.3010	0.0010	-0.9440	oh	1	LIG	-0.528234
3	H1	1.9570	-0.0000	-0.2430	ho	1	LIG	0.306576
4	O2	-0.8840	1.2990	-0.3120	o	1	LIG	-0.724244
5	O3	-0.8840	-1.2990	-0.3130	o	1	LIG	-0.708373
6	O4	0.5130	-0.0010	1.4710	o	1	LIG	-0.724244

REFERENCES

(1) Marcus, Y. Thermodynamics of Solvation of Ions. Part 5. - Gibbs Free Energy of Hydration at 298.15 K. *Journal of the Chemical Society, Faraday Transactions* **1991**, *87* (18), 2995–2999. https://doi.org/10.1039/FT9918702995.
